# Editorial

**Published:** 1844-12-14

**Authors:** 


### THE MEDICAL EXAMINER.

### PHILADELPHIA, DEC. 14, 1844.

We have received several Introductory Addresses, and other publications, some of which will be noticed in our next.

---

What has become of Dr. Copland's Dictionary of Practical Medicine? We noticed the first number on its republication some time ago, (July,) but although it is announced to appear monthly, we have only received "Part I."

---

### CRUVEILHIER'S ANATOMY.

We learn by the Boston Medical and Surgical Journal of the 27th ultimo, that this work has been republished by the Messrs. Harpers, of New York, edited by Professor Pattison. We have had no opportunity of examining the work, as we believe no copies have been sent to the Medical Journals of Philadelphia, although the *penny newspapers* seem not to have been forgotten.

Of Cruveilhier's work it is hardly necessary to speak. It has long been before the Profession. With reference to this edition of it, the editor of the Boston Journal makes the following remarks :

"That this is an admirable work, no one acquainted with the author's writings would think of questioning, and we are gratified in having it accessible to anatomists in this country. But we cannot see that enough has been done by Granville Sharp Pattison, M. D., of New York, to warrant his name appearing, with such an array of titles appended, on the title page, as editor. Part of his Preface, too, is objectionable. It is well known that there is much rivalry existing, at present, between the New York and Philadelphia medical authors, as well as between the large publishers of medical works in the two cities. Much severe language has passed between the former, which will account for the strain of that portion of the Preface to which we have alluded. It is strange language, however for the Preface to a reprint of a scientific work. 'It is very possible,' Dr. Pattison says, 'from the course which these gentlemen reviewers (of Philadelphia) have pursued in reference to the publications which have received the imprimatur of the professors of the University of New York, that the system of anatomy of Cruveilhier, may, when reviewed by them, fare no better than other medical works published by Messrs. Harpers, under the sanction of the Medical Department of New York.' 'It is, however, a matter of very little consequence. *Good wine requires no bush ;* and a good book, if furnished at a low price, must and will always command an extensive sale. New York is the great metropolis of the Union, and must very soon, like London and Paris, however distasteful it may be to those who may have other interests, become the great centre, not only for medical publications, but also of medical education.' Dr. Pattison says also, in the Preface, 'in re-publishing the work, the editor has so restricted himself in the performance of his task, that he feels it can neither add to nor take from his reputation.' He is right in one conjecture, viz., that it will not *add* to his reputation.

We will only repeat, what all cultivators of anatomy now know—that Cruveilhier's system is unexceptional, though perhaps far from being superior to all other works on the same subject. It is a good and desirable book, which will be increasingly valued the more devotedly it is studied. The Messrs. Harpers have, as usual, had their part of the undertaking properly executed. Good paper, good type, &c., are desirable points, which are noticeable in this instance. Some of the cuts, however, are rather indistinct, and draw hard upon the imagination. It is a large octavo, of 907 pages."

Unfortunately for "the publications which have received the imprimatur of the Professors of the University of New York," the criticisms of Philadelphia Journalists have been confirmed by those of other cities in the United States. as well as in Europe. What will the editor of Cruveilheir say of the above notice of his performance, by one who is neither a *Professor* nor a *Philadelphian ?*

